# Protective Effects of Total Saponins of Panax Notoginseng on Steroid-Induced Avascular Necrosis of the Femoral Head In Vivo and In Vitro

**DOI:** 10.1155/2015/165679

**Published:** 2015-01-28

**Authors:** Heng-feng Yuan, Jian-feng Pan, Shuo Li, Chang-an Guo, Shu-hao Liu, Zuo-qin Yan

**Affiliations:** Department of Orthopedics, Zhongshan Hospital, Fudan University, Shanghai 200032, China

## Abstract

This research was designed to investigate the protective effects of TSPN on steroid-induced avascular necrosis of the femoral head (ANFH) and the likely mechanisms of those effects. As an in vivo study, TSPN was shown to be protective against steroid-induced ANFH due to the upregulation of VEGF-A. Furthermore, TSPN attenuated the apoptosis of osteocytes and reduced the expression of Caspase-3 relative to the model group. As an in vitro study, TSPN exerted a concentration-dependent protective effect against apoptosis in MC3T3-E1 cells. Moreover, TSPN (at a dose of 100 *μ*g/mL) significantly reversed the dexamethasone-induced augmentation of Caspase-3 expression and activity. Therefore, our study demonstrated that TSPN had a protective effect against steroid-induced ANFH that was related to the upregulation of VEGF-A and the inhibition of apoptosis and Caspase-3 activation.

## 1. Introduction

Corticosteroid usage is the most common nontraumatic cause of avascular necrosis of the femoral head (ANFH), which can cause interruptions of the blood supply to the bone [1]. If the pathological process remains untreated, more than 70% of cases eventually result in the collapse of the femoral head and subsequent hip destruction [2]. Although the pathogenesis of steroid-induced ANFH remains unclear, it has been found that apoptotic osteocytes play an essential role in the initiation and progression of this disease [3, 4]. Caspase-3, which has been identified as an index of apoptosis, is a critical mediator of the execution phase of cell apoptosis and is involved in many different manners that include the cleaving of DNA repair molecules, skeleton proteins, and extracellular matrix proteins, and the degradation of antiapoptotic proteins [5].

Panax notoginseng is the root of* Panax notoginseng (Burk.) F. H. Chen,* belongs to the family Araliaceae, and has traditionally been used as a highly valued phytomedicine for thousands of years in China. The major components of the total saponins of Panax notoginseng (TSPN) are ginsenoside Rg1, ginsenoside Rb1, notoginsenoside R1, and so forth, and these total saponins have been reported to be the main effective constituents that are responsible for the therapeutic action of this medicine [6]. Multiple reagents of TSPN have been widely used as clinical therapeutic phytomedicines for cardio- and cerebrovascular ischemic diseases due to their effects that include the promotion of angiogenesis, increasing blood flow and improving oxygen supply [7–9]. Recently, it has been reported that TSPN can exert its protective effect through an antiapoptotic mechanism [10, 11].

The aims of the present study were to investigate the protective effect of TSPN on steroid-induced ANFH and to elucidate whether TSPN can protect osteocytes by influencing apoptosis and modulating the expression of Caspase-3 in vitro and in vivo.

## 2. Materials and Methods

### 2.1. Animals and Reagents

Adult male Sprague-Dawley rats (body weight 300–350 g) were raised under controlled temperature (24 ± 2°C) and humidity (55 ± 2%) conditions at the department of laboratory animal science affiliated to Fudan University. Dexamethasone and TSPN (purity: HPLC ≥ 95%) were supplied by Melone Pharmaceutical Co., Ltd. (Dalian, Liaoning, China). MC3T3-E1 cells were supplied by the Cell Bank of the Chinese Academy of Sciences (Shanghai, China). VEGF-A primary antibodies (rabbit anti-rat), Caspase-3 primary antibodies (rabbit anti-rat and rabbit anti-mouse), and the secondary antibodies were supplied by Goodbio Technology Co., Ltd. (Wuhan, Hubei, China). All other chemicals and solvents were of analytical grade.

### 2.2. In Vivo Experiment

#### 2.2.1. Animal Treatment

The rats were randomly divided into the following three groups: a blank control group (*n* = 10), a model group (*n* = 10), and a TSPN group (*n* = 10). The rats in the model and TSPN groups received intragluteal injections of dexamethasone at doses of 50 mg/kg twice per week for 6 weeks. The TSPN group was administered TSPN at doses of 100 mg/kg (i.p.) once per day, and the rats in the blank control and model groups were administered the equivalent volumes of saline solution. The procedures were approved by the Ethical Committee of Animal Experimentation of Fudan University.

#### 2.2.2. Tissue Acquisition and H&E Staining

All rats were killed by anaesthesia overdose, and the femoral heads were fixed in paraformaldehyde for 48 h and decalcified with 10% EDTA solution for 2 weeks. Next, the specimens were embedded in paraffin, cut along the coronal plane of the femoral head, and stained with haematoxylin and eosin (H&E).

#### 2.2.3. Real-Time Polymerase Chain Reaction (RT-PCR)

Total RNA from the femoral head was isolated using Trizol Reagent (Invitrogen) according to the manufacturer's protocol. The integrity and concentration were quantified using a NanoDrop 1000 spectrophotometer (Thermo Scientific). The cDNA was synthesized from the extracted total RNA (500 ng) by reverse transcription according to the instructions of the kit (Fermentas). Real-time quantitative PCR was performed on a 7500 Real-Time PCR System (ABI). Each 10 *μ*L of reaction mixture contained 5 *μ*L of Power SYBR Green PCR Master Mix (ABI), 0.5 *μ*L of specific primers, and 1 *μ*L of cDNA sample. *β*-actin was used as an endogenous control. The mixtures were incubated for 30 min at 16°C, 30 min at 42°C, 5 min at 85°C, and then maintained at 4°C. The relative VEGF-A levels were normalized to *β*-actin expression for each sample in triplicate and are expressed as 2^−ΔΔCT^. The specific primer sequences for VEGF-A were as follows: forward 5′-ACTGGACCCTGGCTTTACTGC-3′, and reverse 5′-TTGGTGAGGTTTGATCCGCATG-3′.

#### 2.2.4. Terminal Deoxynucleotidyl Transferase dUTP Nick End Labelling (TUNEL) Assay

The detections of apoptotic cells in the tissues were performed with an Apoptosis Assay Kit (Roche). Briefly, the paraffin slides were dewaxed and rehydrated, proteinase K was added, and the slides were incubated for 30 min at 37°C. Afterwards, the sections were permeabilized for 20 min and incubated with TUNEL reaction mixture for 60 min at 37°C in the dark. Next, the slides were incubated with 0.03% diaminobenzidine (DAB) for 3–5 min and counterstained with haematoxylin. Nuclei exhibiting intense, homogenous dark brown staining were considered to be indicative of TUNEL-positive cells.

#### 2.2.5. Immunohistochemistry for Caspase-3 and VEGF-A Expression

Briefly, after deparaffinization and rehydration, the sections were immersed in 3% hydrogen peroxide to block endogenous peroxidase activity followed by incubation with normal rabbit serum for 30 min at 37°C. Next, the primary anti-Caspase-3 and anti-VEGF-A antibodies were added to mark their specific antigens, and the sections were incubated overnight at 4°C. The next morning, the sections were incubated with horseradish peroxidase- (HRP-) labelled secondary antibodies for 50 min at 37°C. The sections were then incubated with 0.03% DAB and counterstained with haematoxylin. The negative control sections underwent identical staining with the exception that the primary antibody was excluded.

### 2.3. In Vitro Experiment

#### 2.3.1. Cell Culture

MC3T3-E1 cells (subclone 14) were cultured in cell culture dishes containing alpha minimum essential medium (*α*MEM) supplemented with 10% foetal bovine serum (FBS) and 1% penicillin-streptomycin (PS). The cells were maintained at 37°C in a humidified 5% CO_2_ atmosphere and were subcultured every three days. All cell experiments were repeated three times.

#### 2.3.2. Hoechst 33258 Staining

The MC3T3-E1 cells were seeded onto 96-well culture dishes (5 × 10^4^ cells/well), allowed to attach overnight, and subsequently exposed to dexamethasone (10^−6^ M) and different concentrations of TSPN (50 *μ*g/mL, 100 *μ*g/mL, and 200 *μ*g/mL) for 24 h, 48 h, and 72 h. The cells were washed thrice with PBS, then stained with Hoechst 33258 (0.5 mg/mL) for 15 min, and washed extensively thrice with PBS. The cells were observed under a fluorescent microscope (Olympus BX51, Tokyo, Japan), and the nuclei exhibiting blue fluorescence were considered to indicate probable apoptotic cells.

#### 2.3.3. TUNEL Assay

The TUNEL assays were performed with a TUNEL kit (Beyotime) to label the 3′-end of the fragmented DNA of the apoptotic cells. Briefly, the cells were exposed to dexamethasone (10^−6^ M) and TSPN (100 *μ*g/mL) for 48 h and then fixed in 4% paraformaldehyde, washed thrice with PBS, permeabilized in 0.1% Triton X-100 on ice for 2 min, and subsequently subjected to the TUNEL assay for 1 h at 37°C according to the manufacturer's instructions. The TUNEL-positive nuclei that exhibited green fluorescence under fluorescence microscopy (Nikon Eclipse TI-SR, Tokyo, Japan) were considered to be indicative of apoptotic cells.

#### 2.3.4. Western Blot to Detect Caspase-3 Expression

After treatment, the cells were washed thrice with cold PBS and then lysed in a lysis buffer. The samples were centrifuged at 18,000 ×g for 1 h at 4°C. The supernatants were collected as whole cell lysates. The protein concentrations were estimated, and equal amounts of proteins (10 *μ*g) were resolved by SDS-polyacrylamide gel electrophoresis on 4–20% acrylamide gradient gels and then transferred onto polyvinyldifluoride membranes. The membranes were blocked with a blocking reagent and then incubated with Caspase-3 primary antibody for 1 h at room temperature. After washing, the membranes were incubated with a secondary antibody for 1 h at room temperature.

#### 2.3.5. Caspase-3 Activity Analysis

Caspase-3 activity was measured using a colorimetric assay kit (Beyotime) following the manufacturer's instructions. Briefly, after treatment, the cells were collected and lysed with lysis buffer on ice for 15 min, and the supernatant was then incubated with specific colorimetric peptide substrates (Ac-DEVD-pNA) for 60 min at 37°C. The reaction products were measured at 405 nm with a microplate reader (Eppendorf, Germany). The Caspase-3 activities are expressed as fold increases relative to the baseline observed in the negative controls.

### 2.4. Statistical Analyses

The data are presented as the means ± the standard deviations (S.D.s) and were analysed with one-way ANOVA using SPSS 16.0 (SPSS Inc., Chicago, IL, USA). A *P* value of <0.05 was considered statistically significant.

## 3. Results

### 3.1. Protective Effects of TSPN on Steroid-Induced ANFH

**Figure 1:**
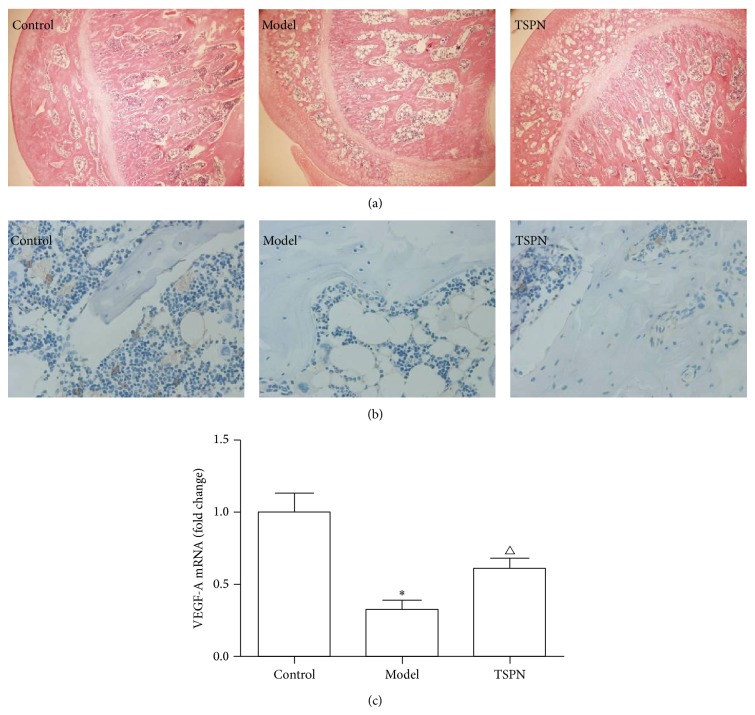
Protective effects of TSPN against steroid-induced ANFH in a rat model. (a) H&E staining of femoral heads of each group (×20). (b) Immunohistochemical staining for VEGF-A (×400). (c) VEGF-A mRNA levels as analysed by real-time PCR. ^*^ *P* < 0.01 compared to the control group; ^∆^ *P* < 0.05 compared to the model group.

H&E staining indicated that the samples obtained from the blank control group exhibited a clear normal ultrastructure of the femoral head (Figure 1(a)). In the model group, the femoral heads of the rats exhibited osteonecrosis with the characteristics of accumulations of fat cells in the bone marrow, irregular and thinner subcartilaginous bone trabeculae with disordered textures, and more frequent appearances of broken bone trabeculae. In the TSPN group, the samples exhibited remarkable protection as evidenced by decreases in the numbers of fat cells and near-normal bone trabeculae.

Immunohistochemical analyses of VEGF-A expression revealed that there were rare VEGF-A-positive areas in the model group, while the TSPN group rats exhibited dramatic increases in the numbers of VEGF-A-positive areas in the femoral head (Figure 1(b)). The RT-PCR results revealed that VEGF-A mRNA was downregulated approximately 3-fold compared to the control group. However, treatment with TSPN significantly improved the VEGF-A mRNA expression (*P* < 0.05; Figure 1(c)).

### 3.2. Antiapoptotic Effects of TSPN in Osteocytes in the Rat Model

**Figure 2:**
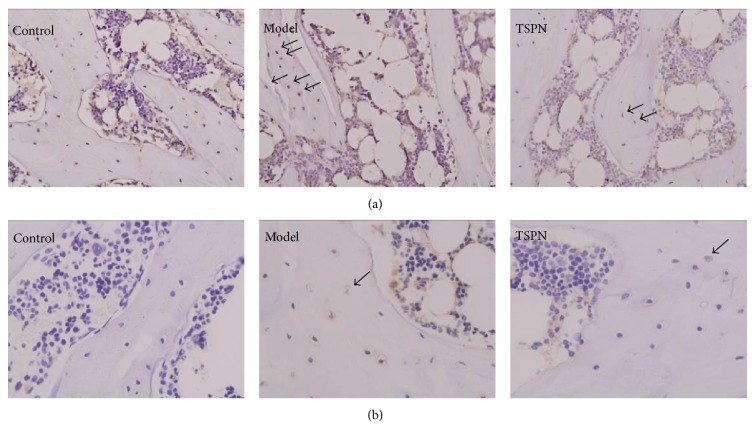
TSPN attenuated apoptosis and reduced Caspase-3 expression in osteocytes in the model of steroid-induced ANFH. (a) TUNEL staining of femoral heads in each group (×200). (b) Immunohistochemical determination of manifestation of Caspase-3 expression (×400). The positive osteocytes are indicated shown with black arrows.

As shown in Figure 2(a), there were minimal numbers of apoptotic osteocytes in the blank control group, while the model group exhibited many apoptotic osteocytes. Moreover, treatment with TSPN decreased the incidence of apoptotic osteocytes.

The immunohistochemical results provided supporting evidence that TSPN reduced the expression of Caspase-3. As shown in Figure 2(b), the Caspase-3-positive osteocytes were buried in the bone trabeculae, and Caspase-3 was primarily expressed in the cytoplasm.

### 3.3. Protective Effects of Different Doses of TSPN in the MC3T3-E1 Cells

**Figure 3:**
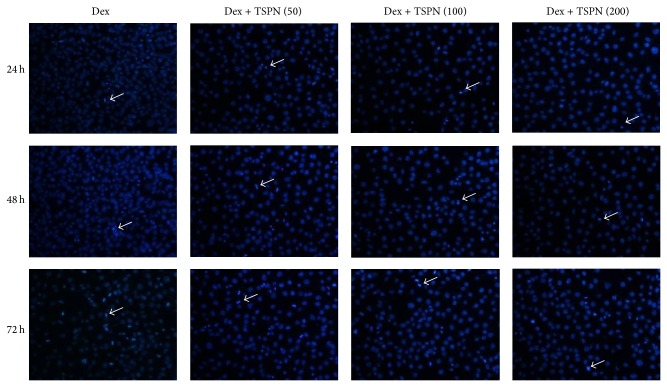
Protective effects of different doses of TSPN in MC3T3-E1 cells. Hoechst 33258 staining of the cells (×200). The positive cells are indicated by white arrows. Dex indicates dexamethasone.

As shown in Figure 3, the cells that were treated with dexamethasone displayed increasing rates of apoptosis with the passage of time. However, incubation with TSPN significantly reduced the rate of apoptosis. Additionally, the high dose of TSPN (200 *μ*g/mL) exhibited improved antiapoptotic ability compared to the lower dose (50 *μ*g/mL).

### 3.4. Caspase-3 Expression and Activity in MC3T3-E1 Cells

**Figure 4:**
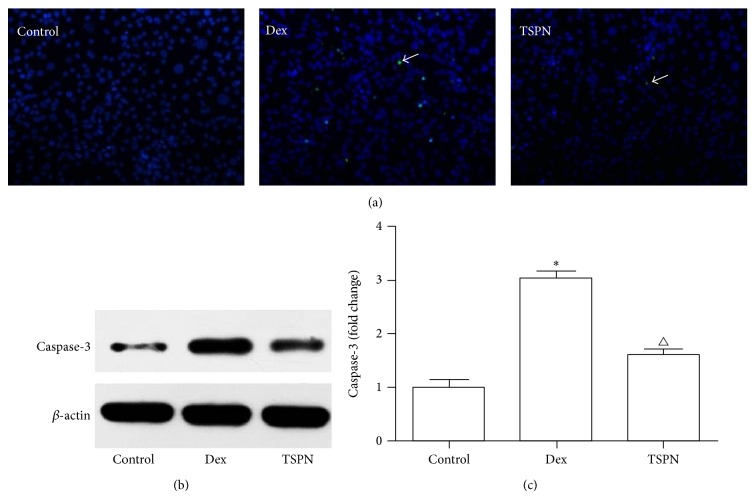
TSPN reduced Caspase-3 expression and activity in MC3T3-E1 cells. (a) TUNEL staining of the apoptotic cells (×200). The positive cells are indicated with white arrows. (b) Western blot for Caspase-3 protein. (c) Caspase-3 activity levels: ^*^ *P* < 0.01 compared to the control group; ^∆^ *P* < 0.01 compared to the Dex (dexamethasone) group.

The TUNEL staining results (Figure 4(a)) revealed that TSPN (100 *μ*g/mL) had antiapoptotic abilities that were consistent with the Hoechst 33258 staining results. The western blot results revealed that, compared to the control group, the Caspase-3 levels in the MC3T3-E1 cells were significantly increased following treatment with dexamethasone. However, following incubation of the cells with TSPN, the Caspase-3 levels significantly decreased (Figure 4(b)). Moreover, the detection of Caspase-3 activity revealed the same protective effect of TSPN (Figure 4(c)). These results illustrated the potential mechanism of the protective effect of TSPN against dexamethasone-induced apoptosis in MC3T3-E1 cells.

## 4. Discussion

Animal models have been widely used to investigate the pathological mechanisms of steroid-induced ANFH. The rat model used in the present study successfully induced osteonecrosis as indicated by histological examinations, and this finding is consistent with a previous report [12]. The typical pathological features of ANFH are characterized by the appearance of empty lacunae and/or ghost nuclei in the lacunae, the accumulation of fat cells in the bone marrow, and irregular and thinner bone trabeculae [13–15]. Specifically, impairments of the blood supply to the bone tissue are thought to be the main factor that initiates this cumulative and irreparable defect [16–18]. TSPN is a traditional and conventional phytomedicine that is used clinically in the treatment of ischemic diseases in China and meets the criterion of the* Pharmacopoeia of the People's Republic of China* (2005). However, there are few reports regarding whether TSPN has protective effects against steroid-induced ANFH.

In the present study, following treatment with TSPN for 6 weeks, the development of dexamethasone-induced ANFH was remarkably prevented. The H&E staining observations revealed that the rats in the TSPN group exhibited nearly normal ultrastructures of the femoral head. Furthermore, we found that TSPN raised VEGF-A expression compared to the model group. These results indicate that TSPN has a potential protective effect on ANFH that is similar to its effects on ischemic diseases.

An early report [3] demonstrated that prominent apoptosis of the osteocytes and cells lining the cancellous bone is present in patients receiving glucocorticoid treatments. In contrast, apoptotic osteocytes are absent or rare in patients with histories of trauma or alcohol abuse, which suggests that steroid-induced ANFH is not a necrotic problem but is actually involved in osteocyte apoptosis [19]. The osteocytes represent the most abundant bone cells, and they descend from osteoblasts and occupy the lacunar space surrounded by the bone matrix. The osteocytes are connected by gap junctions for communication with the bone surface, and once the mechanical information is transmitted into the cells, it could trigger biological cascades such as nitric oxide and prostaglandin E2 with the following Wnt/*β*-catenin signaling, which could also initiate bone remodeling through regulating the osteoblast activity and the osteoclast resorption [3, 20]. Thus the apoptosis of the osteocyte could disrupt its proposed mechanosensory role and prevent functional adaptation of the bone, which could eventually lead to the collapse of the femoral head. However, effective methods for the prevention of osteocyte apoptosis are still lacking. TSPN has previously been reported to be effective in the treatment of focal cerebral ischemia-reperfusion via inhibition of apoptosis [10]; hence, we further investigated its antiapoptotic effects in ANFH.

In the present study, the TUNEL procedure was applied to the femoral head, and positive labelling was confirmed along with the presence of typical signs of nuclear condensation and shrinkage. These results indicate that there were plentiful apoptotic osteocytes in the model group and that TSPN obviously attenuated osteocyte apoptosis. Our results indicate that TSPN elicited antiapoptotic effects in osteocytes in this ANFH rat model.

Dexamethasone has been widely reported to play a pivotal role in the proliferation and differentiation of osteoblasts [21, 22]. Here, we verified that the dexamethasone induced apoptosis in MC3T3-E1 cells and that the rates of apoptosis increased with time. Furthermore, we found that TSPN exerted a dose-dependent protective effect on the MC3T3-E1 cells. The high dose of TSPN exhibited a superior antiapoptotic ability compared to the low dose as indicated by the reduced numbers of Hoechst 33258-positive cells observed on microscopy.

Although the mechanism underlying the apoptosis that is involved in steroid-induced ANFH remains unclear, it has been found that Caspase-3 might play an important role in the development of this disease. A recent study demonstrated that the apoptotic process in steroid-induced ANFH develops with the upregulation of Caspase-3 [23]. A previous in vitro study showed that glucocorticoids can elevate Caspase-3 levels via both endogenous and exogenous apoptosis pathways in MC3T3-E1 cells [24], and osteocytes and osteoblasts transfected with calbindin-D28k (an apoptosis inhibiting protein) exhibit suppressed steroid-induced Caspase-3 activity [25]. In the present study, we tested the expression and activity of Caspase-3 with different methods. We found that Caspase-3 was strongly elevated in the model group compared to the blank control group rats, and TSPN effectively reduced this expression. In our in vitro study, we confirmed the protective effect of TSPN that was mediated by reduced expression and activity of Caspase-3, which indicates that the protective mechanism of TSPN might correlate with the inhibition of Caspase-3.

Taken together, these results show that TSPN has a positive protective effect on steroid-induced ANFH that is mediated by reductions in the rate of apoptosis in osteocytes due to the inhibition of Caspase-3 activation. TSPN might be a useful protective phytomedicine for patients who are in need of corticosteroid treatments and are at risk of developing ANFH. However, further in vitro and in vivo studies should be performed to support the clinical application of TSPN.
